# Retracted: Identification of the Novel Methylated Genes' Signature to Predict Prognosis in INRG High-Risk Neuroblastomas

**DOI:** 10.1155/2022/9846323

**Published:** 2022-12-28

**Authors:** Journal of Osteoporosis

*Journal of Oncology* has retracted the article titled “Identification of the Novel Methylated Genes' Signature to Predict Prognosis in INRG High-Risk Neuroblastomas” [1] due to concerns that the peer review process has been compromised.

Following an investigation conducted by the Hindawi Research Integrity team [2], significant concerns were identified with the peer reviewers assigned to this article; the investigation has concluded that the peer review process was compromised. We therefore can no longer trust the peer review process, and the article is being retracted with the agreement of the Chief Editor.

The authors agree to the retraction.

## References

[1] Liu Z., Li C. (2021). Identification of the Novel Methylated Genes’ Signature to Predict Prognosis in INRG High-Risk Neuroblastomas. *Journal of Oncology*.

[2] Ferguson L. (2022). Advancing Research Integrity Collaboratively and with Vigour. https://www.hindawi.com/post/advancing-research-integrity-collaboratively-and-vigour/.

